# Consumer awareness, trust and cultural challenges in the use of child car seats in Iran: Current status and improvement strategies

**DOI:** 10.1371/journal.pone.0351222

**Published:** 2026-06-22

**Authors:** Ebrahim Zarepour Nasirabadi, Shiva Yazdani, Bahram Shirini, Mirbahador Yazdani

**Affiliations:** 1 Department of Business Administration, Faculty of Governance, Shahed University, Tehran, Iran; 2 Health and Rehabilitation Sciences, University of Western Ontario, London, Canada; 3 Faculty of Engineering, University of Bonab, Bonab, Iran; Khoy University of Medical Sciences, IRAN, ISLAMIC REPUBLIC OF

## Abstract

**Background:**

Injuries that occur on the road are greatly affecting young children across the globe. Learning about the myths future parents may have and the barriers they may face is important. This study aimed to assess parental awareness, trust, cultural factors, and barriers related to the use and correct use of child car seats among parents of young children in Iran.

**Methods:**

This cross-sectional study included approximately 750 parents of children aged 1–3 years in Tabriz, Iran. The data were collected via a structured questionnaire conducted at 19 public health centers. An expert panel confirmed content validity (CVI = 0.89; CVR range: 0.71–1.00), and internal consistency was acceptable (Cronbach’s alpha = 0.82). Data were analyzed using descriptive statistics and inferential methods, including chi-square tests, t-tests, ANOVA, and regression models.

**Results:**

Although 92% of participants owned a car, only 47% owned a child car seat, and 55% reported rarely or never using one. Awareness of age and weight recommendations was high (85%), but knowledge of correct installation was lower (62%). Car seat use was significantly lower during city trips (45%) compared to out-of-city trips (82%) (d = 1.25). Consistent fastening of the child’s seatbelt during city trips was 28%. Significant predictors of fastening the seatbelt include Trust (β = 0.45), Education (β = 0.30), and Risk Perception. Major barriers included high cost (62%), lack of awareness (55%), and cultural practices such as holding children on laps (50%).

**Conclusion:**

High vehicle ownership alone does not ensure child safety. Multilevel interventions combining skills-based education, affordability, product quality assurance, social norm change, and policy enforcement are needed to improve both the use and correct use of child car seats.

## Introduction

Road traffic accidents remain one of the leading causes of injury and mortality worldwide [1,2], posing a particularly serious threat to children [3]. They represent the fourth leading cause of death among children aged 5–9 years, the third among those aged 10–14 years, and the first among adolescents aged 15–17 years [4]. According to global and regional reports, children are among the most vulnerable road users due to their physical fragility and limited ability to protect themselves during collisions [5]. When traveling in private vehicles, inadequate restraint systems substantially increase the risk of severe injury or death for young passengers [6]. In many low- and middle-income countries, rapid motorization has not been accompanied by proportional improvements in child road safety practices, making traffic-related injuries a major and preventable public health concern [7,8].

The use of appropriate child restraint systems, including child car seats, is one of the most effective measures for reducing injury severity and mortality among child passengers [9]. Properly installed and correctly used child car seats significantly decrease the risk of fatal and non-fatal injuries by distributing crash forces and preventing ejection during collisions. International guidelines strongly recommend age- and weight-appropriate child restraints for all vehicle journeys. Despite this strong evidence, compliance with child car seat recommendations remains inconsistent in many settings, particularly for short trips and in urban environments [10].

Parents determine if and how often child car seats are used, and how often used in the correct manner. Factors such as knowledge of guidelines regarding safety, risk and protective actions, and trust in child safety seats and safety guidelines impact behavioral response [11]. Parents who have a higher perceived trust in the effectiveness of the safety seats are more likely to use them. Misconceptions about accident risk, particularly during short distance travel, can adversely affect use of seats. Parents’ confidence in the safety of car seats available to them and the trustworthiness of the safety guidelines also impacts decision making [12].

Beyond the parent’s individual factors, there are cultural norms, social factors, and policies that impact car seat use [13–15]. In some cultures, it is considered more caring to hold children during travel, which goes against modern safety recommendations [16]. Family pressure, social disapproval, and lack of enforcement may also further inproper usage. Weak policies, low enforcement, high prices of car seats, and poor recognition of policy importance can also impact lack of usage. Thus, use of car seats can be viewed as a behavior that is impacted by social, individual, and structural factors [17].

Recent studies have consistently shown that the use of child restraint systems remains suboptimal, particularly in settings where motorization has increased more rapidly than road safety culture [18]. Survey-based and observational research indicates that although many parents recognize the general importance of child safety, actual usage rates of child car seats are relatively low [19]. Commonly reported barriers include limited knowledge of correct usage, high cost of certified child seats, and the absence or weak enforcement of child restraint regulations. These studies emphasize that awareness alone does not guarantee proper adoption of child car seats, especially in everyday driving situations [20,21].

More recent research has shifted from merely documenting prevalence toward examining determinants of parental behavior, including awareness, attitudes, and trust [20]. Several studies have found that higher parental education is associated with better knowledge of age- and weight-appropriate child restraints, as well as greater likelihood of use [22]. However, even among parents with adequate knowledge, inconsistent use has been observed, particularly during short trips [23]. This suggests that risk perception and attitudes such as beliefs that short trips are safe or that holding a child is protective play a critical role in decision-making.

Regional studies highlight considerable variability and generally low adherence to child car seat recommendations. For example, a study conducted in Gorgan, Iran, reported that approximately 80% of parents never used a child safety seat, while only 13% reported consistent use. Notably, more than 93% of parents supported making child car seat use mandatory, suggesting a gap between positive attitudes and actual behavior [24]. In neighboring countries, similarly concerning patterns have been observed. In the United Arab Emirates, car seat use among children younger than 24 months was reported to be less than 20%, and improper usage was common even among owners [25]. In Saudi Arabia, although about 50.6% of parents reported always using a car seat, one-third (33.5%) reported never using one [26].

Another important body of literature has highlighted the role of trust and confidence in child restraint systems. Parents who trust the effectiveness of child car seats and the credibility of safety recommendations from health professionals are more likely to use them consistently [27]. Conversely, doubts about the quality of available products, uncertainty regarding installation, and lack of confidence in proper use can discourage adoption. Studies also report that perceived complexity of installation and adjustment, as well as children’s resistance to sitting in car seats, contribute to partial or incorrect use, such as placing the child in the seat without fastening the restraint [28].

Although some studies in Iran have explored general patterns of child car seat use, there remains limited evidence regarding the underlying cognitive, cultural, and trust-related determinants influencing parental behavior. Furthermore, few studies have assessed how cultural norms and beliefs interact with knowledge and policy awareness to influence child safety practices [24].

Given these gaps, the present study aims to assess the level of knowledge, trust, and behavioral practices related to child safety seat use among parents of young children in Tabriz, Iran. By identifying policy-related, individual, and cultural determinants of child car seat utilization, this research seeks to provide evidence to inform targeted interventions and guide policymakers in developing context-specific strategies to improve child passenger safety.

## Methods

The current study uses a cross-sectional description and analytical technique to assess knowledge, trust, and cultural barriers with respect to the use of child safety seats, with the sample being Tabriz parents of young children. Tabriz is a major metropolitan city in northwestern Iran. Increased motorization has resulted in more road traffic risks due to rapid urbanization in the city. Culturally, family-centered values are strong, and traditional childcare practices such as holding children during travel remain common and may influence safety behaviors. In addition, variability in socioeconomic status, access to safety equipment, and public awareness of child passenger safety may affect the adoption and correct use of child car seats.

The study’s instrument was a questionnaire developed by the researchers and administered in person. The study sample included parents, i.e., mothers and fathers, of children aged 1–3 years, who visited 19 public health clinics in Tabriz, the provincial capital of Tabriz, for routine health surveillance of children (Data collection was conducted from April 1, 2025 to August 1, 2025). The sample included about 750 parents. For this control of the sample’s representativeness, a control of a balanced distribution of the sample was set for mothers and fathers in the selected health centers, and it was recognized in advance for control purposes that exactly balanced control of the sample. The sample size was calculated using the standard formula for estimating a population proportion in cross-sectional studies, assuming a 95% confidence level (Z = 1.96), an expected prevalence of 20% based on previous regional findings, and a margin of error of 3%, which yielded a minimum required sample of 683 participants; The final sample size was increased to 750 parents to ensure adequate statistical power.

A convenience sampling approach was used, whereby eligible parents who were present at the health centers during the data collection period were invited to participate. Only one parent per child was surveyed, unless both were present and consented to participate independently. The inclusion and exclusion criteria applied in this study are summarized in Table 1.

**Table 1:** Inclusion and exclusion criteria.

Category	Criteria
**Inclusion Criteria**	Participants were biological or legal parents (mother or father) of a child aged 1–3 years.
	Participants had brought their child to the selected health center for routine check-up services.
	At least one parent in the household owned or regularly used a private car, allowing the possibility of child car seat use.
	Participants were able and willing to provide informed consent.
	Participants were able to understand and respond to the questionnaire in Persian.
**Exclusion Criteria**	Participants were caregivers other than parents (e.g., grandparents, relatives, or nannies).
	The child had a medical condition that limited typical car seat use (e.g., severe mobility constraints), based on parent report.
	Participants had previously taken part in the study, to prevent duplicate responses.
	Participants were unable or unwilling to complete the questionnaire (e.g., due to time constraints or other reasons).

### Data collection procedure

Data collection took place within designated child health centers across Tabriz. A convenience sampling approach was employed to recruit eligible parents attending public health centers during the study period because these centers serve as primary and routinely utilized child healthcare facilities, providing access to a broad cross-section of families from different socioeconomic and geographic backgrounds; although probability sampling methods such as random or cluster sampling may offer stronger representativeness, their implementation was not feasible due to the absence of a comprehensive sampling frame of eligible parents, logistical constraints, and limited resources. After confirming eligibility, trained interviewers explained the study purpose and obtained written informed consent. The structured questionnaire was then administered in person, either through self-completion or interviewer assistance for parents needing help with reading or comprehension. Each interview lasted approximately 10–15 minutes. To ensure privacy and reduce response bias, parents completed the questionnaire in a quiet area of the health center away from clinical staff.

### Research instrument

A structured questionnaire was developed containing several sections. These fields explained the basic demographic data of parents, such as age, sex, academic qualifications, job title, if they held a car, and the number of children they had. Additionally, the questionnaire measured the parents’ knowledge of child car seat safety, such as knowing the age and weight appropriate car seat for a child, and the proper ways of securing a child into a car seat, and the ways a car seat can prevent injuries. Other aspects of the person’s feelings were also measured, such as the child car seat’s perceived safety, trusts the safety guidelines, and thinks child car seats are effective. The other barriers explored by the questionnaire was the perceived social norms and cultural practices, the affordability of a car seat, the child’s comfort, the child’s safety seat, the habitual behaviors of adult. The majority of the questions in the survey were scaled along a Likert scale, or framed as multiple choice questions, and a few were left open-ended to get more qualitative data.

Prior to full implementation, the questionnaire underwent a rigorous validation process. Content validity was evaluated through expert review by a multidisciplinary panel of seven specialists with expertise in pediatrics, public health and maternal–child health, road safety and injury prevention, health education and promotion, and epidemiology. The experts assessed the instrument in terms of relevance, clarity, comprehensiveness, and cultural appropriateness. Based on their feedback, several items were revised for improved clarity, redundant questions were removed, and culturally specific examples were incorporated. Content validity of the questionnaire was quantitatively assessed using the Content Validity Ratio (CVR) and the Content Validity Index (CVI) based on evaluation by seven experts, with item-level CVR values ranging from 0.71 to 1.00 (retaining items above the Lawshe minimum acceptable value of 0.62 for seven experts) and item-level CVI values ranging from 0.83 to 1.00, resulting in a scale-level CVI (S-CVI/Ave) of 0.89, indicating excellent content validity. Subsequently, the revised questionnaire was pilot tested with 20 parents who met the study inclusion criteria but were not included in the final sample. Data from the pilot study were used to evaluate internal consistency, yielding a Cronbach’s alpha of 0.82 for the total scale. The awareness, trust, and cultural barriers subscales demonstrated acceptable to good reliability, with Cronbach’s alpha values of approximately 0.78, 0.80, and 0.83, respectively, indicating good internal consistency of the instrument.

### Data analysis

Data were analyzed using SPSS software (version 23). Descriptive statistics, including frequencies, percentages, means, and standard deviations, were used to summarize participants’ demographic characteristics and key study variables. Inferential statistical analyses were conducted to examine associations between demographic factors and levels of awareness, trust, and perceived cultural barriers related to child car seat use. Chi-square tests and independent sample t-tests were applied as appropriate, and logistic regression analysis was used to identify predictors of awareness, trust, and reported barriers. A significance level of p < 0.05 was considered statistically significant.

### Ethical considerations

Ethical approval for the study was obtained from the relevant institutional review board. Participation in the study was entirely voluntary, and informed consent was obtained from all participants prior to data collection. Responses were collected anonymously, and no identifying information was recorded. Participants were informed of their right to withdraw from the study at any time without any consequences. The study was conducted in accordance with ethical standards for research involving human participants (Ethical Code: IR.TBZ.REC.1404.716). Details of the study questionnaire used to assess child car seat use are shown in Table 2. The questionnaire was translated from English into Persian using a forward–backward translation method. The final Persian version was reviewed for clarity and cultural appropriateness before data collection. Table 2 presents the English version of the questionnaire, while data were collected using the validated Persian version.

**Table 2:** Study questionnaire on child car seat use.

Section	Item No.	Question/ Statement	Response Options/ Scale
**Section 1: Demographic and Background Information**	1	Parent’s gender	Mother/ Father
	2	Education level	Primary/ Secondary/ High school diploma/ University degree (Associate/Bachelor)/ Graduate degree
	3	Does your household own or regularly use a private car?	Yes/ No
	4	Do you currently own a child car seat?	Yes/ No
	5	How often do you use a child car seat when traveling by car?	Always/ Often/ Sometimes/ Rarely/ Never
**Section 2: Awareness of Child Car Seat Safety**	6	I know the recommended age for using a child car seat.	Likert scale (1–5)
	7	I know the recommended weight and height limits for child car seats.	Likert scale (1–5)
	8	I am aware of how to properly install a child car seat in the car.	Likert scale (1–5)
	9	I understand that using a child car seat significantly reduces injury in car accidents.	Likert scale (1–5)
**Section 3: Trust in Child Car Seats and Safety Guidelines**	10	I trust that child car seats provide real protection in accidents.	Likert scale (1–5)
	11	I trust the safety recommendations provided by health professionals regarding car seat use.	Likert scale (1–5)
	12	I trust that car seats sold in Iran meet international safety standards.	Likert scale (1–5)
	13	I believe child car seat rules and guidelines are necessary and justified.	Likert scale (1–5)
**Section 4: Cultural, Social, and Practical Challenges**	14	In my family or community, it is common to hold children on laps instead of using a car seat.	Likert scale (1–5)
	15	People around me (family, relatives, friends) do not think car seats are necessary.	Likert scale (1–5)
	16	I feel judged or criticized for using a car seat.	Likert scale (1–5)
	17	I think car accidents are unlikely to happen to my family.	Likert scale (1–5)
	18	The cost of car seats is a major barrier for my family.	Likert scale (1–5)
	19	Using a car seat is inconvenient during short trips.	Likert scale (1–5)
	20	Car seats available in the market are not affordable.	Likert scale (1–5)
	21	My child resists sitting in the car seat.	Likert scale (1–5)
**Section 5: Child Car Seat Use During Travel**	22	Do you use a child car seat for trips within the city?	Always/ Often/ Sometimes/ Rarely/ Never
	23	When using a child car seat, do you always fasten your child’s seatbelt?	Yes, always/ Sometimes/ No, never
	24	Do you use a child car seat for trips outside the city (e.g., long-distance travel)?	Always/ Often/ Sometimes/ Rarely/ Never
	25	When using a child car seat on trips outside the city, do you always fasten your child’s seatbelt?	Yes, always/ Sometimes/ No, never
**Section 6: Improvement Strategies**	26	Providing affordable or subsidized child car seats would increase usage.	Likert scale (1–5)
	27	More media campaigns are needed to raise awareness among Iranian parents about child car seat safety.	Likert scale (1–5)
	28	I would use a car seat more often if it was easier to install and use.	Likert scale (1–5)
	29	I would use a car seat more often if family members supported the practice.	Likert scale (1–5)
	30	Workshops or demonstrations at health centers would help parents understand correct usage.	Likert scale (1–5)
	31	Stricter enforcement of car seat laws would lead to greater use.	Likert scale (1–5)
**Section 7: Open-Ended Questions**	33	What do you think is the biggest obstacle to using a child car seat in your family or community?	Open-ended
	34	What changes would most help parents in Iran use car seats more regularly?	Open-ended
	35	Any additional comments or suggestions?	Open-ended

## Results

Demographic data of the respondents regarding the objectives of the study illustrated in Table 3 includes the level of awareness and trust, cultural barriers and their behaviors regarding the use of child car seats, the frequency of use, and the perceptions. The sample offered comprised of equal number of respondents who were parents, predominantly in possession of cars, although in this aligned with previous research the parents with less than fifty percent of the sample claimed owning a car seat and the majority of them conveyed not using it, which denotes a discrepancy between the ownership of a car and the recommended practices of child safety.

**Table 3:** Descriptive overview of participant characteristics and child car seat–related variables.

Category	Variable/ Theme	Result (Mean ± SD or n (%))
**Demographics**	Mothers	390 (52%)
	Fathers	360 (48%)
	Car ownership	690 (92%)
	Child car seat ownership	353 (47%)
	Frequency of use – Always	120 (16%)
	Often	105 (14%)
	Sometimes	113 (15%)
	Rarely/Never	412 (55%)
**Awareness**	Awareness of age/weight limits	638 (85%); 4.2 ± 0.6
	Knowledge of installation	465 (62%); 3.6 ± 0.8
	Understanding safety benefits	585 (78%); 4.3 ± 0.7
	General awareness score	3.7 ± 0.6
**Trust**	Trust in effectiveness	623 (83%); 4.1 ± 0.6
	Trust in professionals	645 (86%); 4.2 ± 0.5
	Trust in local seat quality	533 (71%); 3.6 ± 0.7
	General trust score	3.9 ± 0.5
**Cultural Barriers**	Holding child on lap (city trips)	675 (90%)
	Others think car seats unnecessary	533 (71%)
	Feel judged for using seat	375 (50%)
	Believe accidents unlikely	510 (68%)
**Usage Patterns**	Car seat use within city	338 (45%)
	Seatbelt fastened within city	210 (28%)
	Holding child in arms (city)	675 (90%)
	Car seat use outside city	615 (82%)
	Seatbelt fastened outside city	660 (88%)
**Improvement Preferences**	Subsidized seats	690 (92%)
	Media campaigns	660 (88%)
	Workshops at health centers	630 (84%)
	Stricter enforcement	585 (78%)
**Open-Ended Themes**	High cost	465 (62%)
	Lack of awareness	413 (55%)
	Cultural habit of holding child	375 (50%)
	Difficulty of installation	300 (40%)
	Concerns about effectiveness	225 (30%)

Awareness was concentrated in the area of the safety that is recommended, barriers to its use and the age-appropriate infant and child safety seat in the car, yet the actual knowledge of the appropriate safety seat was lacking this established a difference between awareness of safety and knowledge of safety. Trust in the effectiveness of car seats and health professional guidance was strong, yet trust in the quality of locally available car seats was weaker, which could affect purchasing and usage decisions.

Cultural and social barriers in the society were very instrumental in this regard; a number of parents during a trip to the city held their child on their lap which was a customary practice; this was due to the very low perception of necessity to use a car seat, and their underestimation of the risk of an accident. There were substantial differences in the use of a car seat in the city as opposed to having a long trip. More parents secured their children in a car seat and also fastened their own seat belts in the car. Most of the participants strongly supported the idea of improving the use of car seats which included the provision of free car seats, education and awareness campaigns, and more rigid laws that encourage the use of a car seat. The responses highlighted obstacles such as high cost, lack of awareness, entrenched habits, installation challenges, and doubts about effectiveness.

**Fig 1:**
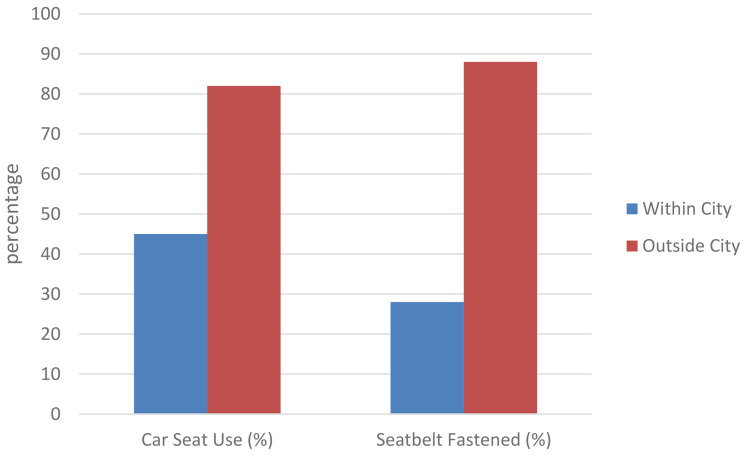
Comparison of child car seat use and seatbelt fastening within and outside the city.

Fig 1 shows significant variations in child seat use and seat belt use, with compliance being significantly higher on out-of-town trips than on city trips. While only about 45% of parents reported using a child seat on city trips, this proportion increased significantly to 82% for out-of-town trips. A similar pattern was observed for seat belt use. This difference reflects differences in perceived risk. Parents may associate highways and long trips with higher speeds, heavier traffic, and more severe crashes, thereby prompting more protective behaviors. In contrast, short city trips are often perceived as routine and low-risk trips, leading to complacency and reduced adherence to safety measures. In addition, practical factors including frequent stops, time constraints, and the perceived inconvenience of repeatedly fastening and unfastening belts may discourage their continued use in urban settings. These findings suggest that interventions should specifically target misconceptions about short-haul trips and emphasize that the risk of an accident exists regardless of the length or location of the trip.

**Fig 2:**
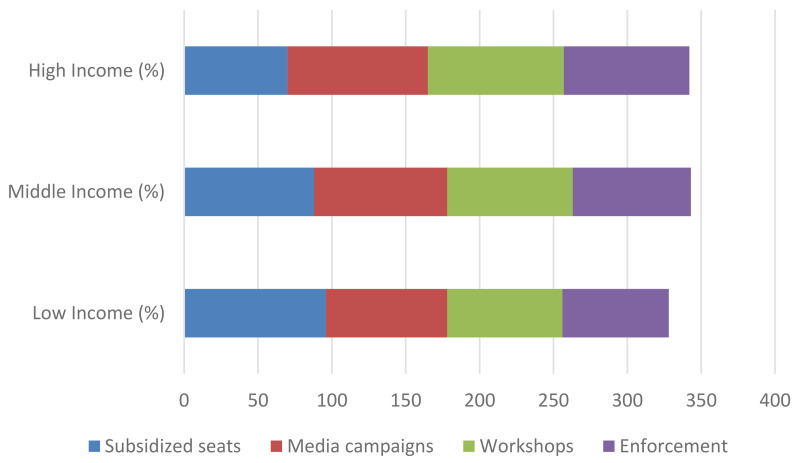
Preferred child car seat improvement strategies by household income level.

Fig 2 illustrates parental preferences for strategies to increase child car seat usage across different income levels. Subsidized car seats and media campaigns received the highest overall support. Lower-income families favored subsidies, while higher-income families leaned towards enforcement methods. Workshops and educational interventions enjoyed consistent support across all income levels, indicating a universal appreciation for practical guidance.

**Fig 3:**
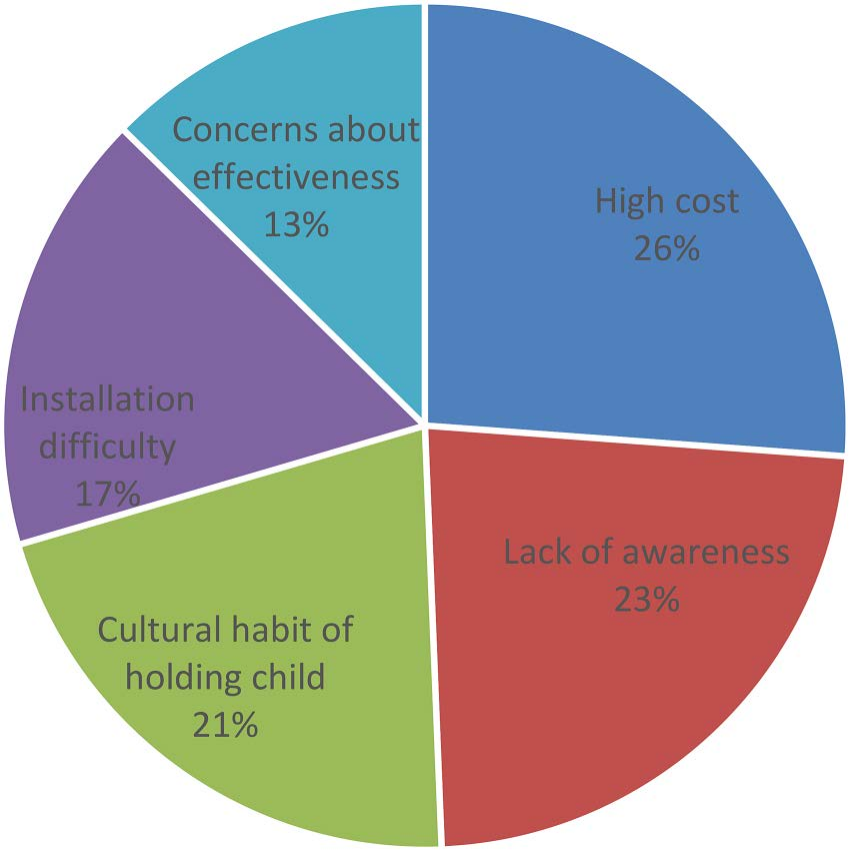
Reported barriers to child car seat use among parents.

Fig 3 highlights the main barriers to child car seat use, revealing that high cost is the predominant obstacle. Lack of awareness and the cultural practice of holding children in caregivers’ arms are also significant. Additional practical challenges, including installation difficulty and effectiveness concerns, were noted but less frequently. These results illustrate the complex influence of economic, cultural, and informational factors on child car seat usage.

Child car seat use appears to be more influenced by cognitive factors, financial considerations, contextual factors, as well as people’s attitudes, than by demographic factors alone. This is evidenced by Relationships across the child car seat data pertaining to child car seat use (Table 4). Basic demographic information revealed that there were no consistent differentials in child car seat use in relation to the sex of the parent or the age of the parent. The data revealed that the level of education of the respondent was an important variable that had consistently been associated with greater awareness of available information and guidelines pertaining to child car seat use as well as an increased level of trust in the recommendations of child car seat use guidelines for safety. This indicated that the level of education was fundamental in assessing risk as well as having certain confidence in taking preventive actions that would be required to be proactive in the use of child car seats. The data showed that trust in the car seats and the guidelines was associated with greater car seat use as well as correct seat belt fastening. This emphasized trust as a variable that explained the correlation between knowledge and behavior. The data indicated that while socioeconomic status had an inconsistent influence on the actual use of child car seats, it did influence the support for programs that provided financial assistance. This risk perception shaped on the use of child car seats, more markedly for brief use in the urban context as there was greater car seat use when the parents perceived a greater risk. The variance in car seat compliance between urban and out-of-city trips underscores the importance of situational norms and perceived severity in safety practices.

**Table 4:** Inferential statistical models examining predictors of child car seat use and safety behaviors.

A.Bivariate Analyses										
Analysis	Variables			Test Statistic			df	p-value		Effect Size
Chi-square	Gender × Car seat ownership			χ² = 0.58			1	0.44		—
Independent t-test	Age (Mothers vs. Fathers)			t = –1.56			748	0.12		Cohen’s d = 0.11
One-way ANOVA	Awareness score by Education level			F = 14.2			4,745	<0.001		η² = 0.07
Pearson Correlation	Education level × Trust score			r = 0.38			—	<0.001		Moderate positive
**B. Linear Regression Model Predicting Child Car Seat Use Frequency**										
**Dependent variable: Car seat use score** **Model statistics: R² = 0.38, Adjusted R² = 0.37, F (3,746) = 152.3, p < 0.001**										
**Predictor**	**β (Standardized)**			**SE**			**t**	**p-value**		**95% CI**
Trust score	0.45			0.04			10.8	<0.001		0.37–0.53
Education level	0.30			0.05			6.9	<0.001		0.21–0.39
Socioeconomic status	0.18			0.06			1.89	0.06		–0.01–0.24
**C. Logistic Regression Predicting Car Seat Use on Short Trips**										
**Dependent variable: Use on short trips (Yes/No)** **Model fit: χ² (3) = 84.6, p < 0.001, R² = 0.29**										
**Predictor**	**β**			**OR**		**95% CI for OR**				**p-value**
Risk perception	0.68			1.97		1.42–2.74				<0.01
Social support	0.55			1.73		1.10–2.43				<0.05
Education level	0.75			2.11		1.54–2.90				<0.001
**D. Paired t-test Comparing City vs. Out-of-City Use**										
**Comparison**	**t**			**df**		**p-value**				**Cohen’s d**
Within city vs. Outside city	–15.8			749		<0.001				1.25
**E. Logistic Regression Predicting Seatbelt Fastening**										
**Dependent variable: Seatbelt fastening (Yes/No)** **Model fit: χ² (3) = 96.4, p < 0.001, R² = 0.33**										
**Predictor**	**β**			**OR**		**95% CI**				**p-value**
Ease of use	0.75			2.12		1.41–3.01				<0.01
Risk perception	1.25			3.49		1.65–4.58				<0.05
Trust	0.95			2.58		1.42–3.37				<0.01
**F. Linear Regression Predicting Support for Subsidized Car Seats**										
**Dependent variable: Support for subsidies** **Model statistics: R² = 0.41, Adjusted R² = 0.40, F (3,746) = 173.5, p < 0.001**										
**Predictor**	**β (Standardized)**		**SE**	**t**		**p-value**				**95% CI**
Trust	0.56		0.05	12.1		<0.001				0.47–0.65
Education level	0.45		0.06	7.8		<0.01				0.32–0.58
Income	0.38		0.07	6.4		<0.01				0.24–0.52
**G. Chi-square Test: Support for Subsidies by Income Level**										
**Test**		**χ²**			**df**				**p-value**	
Income × Support for subsidies		22.5			4				<0.001	

Moreover, analyses on support for improvement strategies found that lower-income parents favored subsidized car seat programs, with education and trust reinforcing support for such policies. The overall findings suggest that interventions addressing child car seat use should be multifaceted, integrating education, trust-building, economic support, and context-specific messaging for effective promotion of safe practices.

## Discussion

Previous research shows that there is a high percentage (92%) of vehicle ownership among parents but a low percentage (47%) of child car seat ownership and usage. The parents who do own child car seats say they do not use them often. This trend shows that having access to private vehicles is not directly correlated with safe child passenger practices. Other research has shown similar low usage of child restraints when there is a high volume of car ownership, particularly in low- and middle- income countries [29]. This situation illustrates that having a vehicle does not improve child safety in a car. Other behavioral issues and limitations (cultural and economic) are to blame. Research suggests that increased access to vehicles alone does not improve child safety. Without safety education programs, consistent enforcement, and affordable child restraints, greater vehicle access may actually increase the risk of injury [30]. This shows that parents are not changing their behavior when it comes to child safety. There are other variables not directly related to vehicle access that are restricting child safety practices.

Our findings are consistent with previous studies conducted in Iran and neighboring countries, which report low and inconsistent use of child car seats despite generally positive attitudes toward child safety. In a study from Gorgan, Iran, approximately 80% of parents reported never using a child safety seat and only 13% reported consistent use, highlighting a substantial gap between awareness and actual practice [24]. Similarly, studies from the United Arab Emirates [25] and Saudi Arabia [26] have documented low or inconsistent use, particularly during short trips, even among parents who own car seats.

Although parents in this study demonstrated relatively high awareness of age and weight recommendations (85%) and injury-prevention benefits (78%), their knowledge of correct installation was notably lower (62%), and the overall awareness score was only moderate. This gap between conceptual knowledge and practical competence has been widely documented in the literature. Several studies have found that parents often understand that child car seats are important, yet lack the technical skills needed for correct installation and consistent use [31].

One of the most important findings of this study is the central role of trust in shaping both the use and correct use of child car seats. While trust in health professionals was high (86%), trust in the quality of locally available car seats was lower (71%). Importantly, trust emerged as a significant predictor of car seat use (β = 0.45), seatbelt fastening (β = 0.95), and support for subsidization policies (β = 0.56). These results align with previous research demonstrating that trust acts as a mediator between knowledge and behavior [15]. Parents who trust safety recommendations and believe in the effectiveness of child restraint systems are more likely to translate awareness into action. Conversely, doubts about product quality or safety standards can undermine compliance, even among parents who recognize the theoretical importance of child seats.

Cultural and social factors emerged as powerful barriers to child car seat use in this study. The overwhelming prevalence of holding children on laps during city trips (90%), combined with the perception that car seats are unnecessary (71%) and feelings of social judgment for using them (50%), indicates that unsafe practices have become socially normalized. Similar cultural patterns have been described in previous studies, where traditional caregiving beliefs and social expectations discouraged the adoption of child restraint systems [32–34].

These findings highlight that child safety decisions are socially negotiated, rather than purely individual choices. Parents may experience tension between safety knowledge and the desire to conform to family or community norms. Our results support calls in the literature for community-level interventions, including normalization of car seat use through media representation, role modeling, and consistent enforcement. Addressing social pressures and reshaping cultural expectations may be essential for achieving sustained improvements in child passenger safety.

A key finding of this study is the clear difference in child car seat use between urban, short-distance trips (45%) and out-of-city travel (82%). This difference was substantial and was supported by a very large effect size (d = 1.25). Furthermore, 68% of parents believed that car accidents were unlikely, indicating a systematic underestimation of risk during routine travel. Previous studies have demonstrated that risk perception, rather than objective exposure, is a dominant driver of safety behavior. Parents often reserve protective practices for situations they perceive as dangerous, such as highways or long-distance travel, while neglecting them during everyday trips [35]. Cognitive biases, including familiarity bias and optimism bias, may lead parents to underestimate risk in routine contexts, resulting in inconsistent safety behaviors. Our findings support this interpretation and suggest that distance is not the determinant of safety behavior; perceived risk is. This highlights the need for interventions that explicitly challenge misconceptions about short trips and emphasize that crash risk exists regardless of distance or familiarity.

Although some parents reported placing their child in a car seat during city trips, only 28% fastened the child’s seatbelt. Logistic regression analysis identified ease of adjustment, trust in car seat safety, and risk perception as significant predictors of correct seatbelt fastening. Partial compliance such as using a car seat without fastening the restraint may create a false sense of safety, potentially increasing risk rather than reducing it. Studies have consistently shown that misuse rates of child restraint systems are high, even in settings where overall usage is moderate, and that complexity of installation and adjustment is a major contributor to misuse [36]. Our results reinforce the importance of usability and design simplicity, suggesting that technical barriers can undermine safety even when motivation exists. Improving user-centered design and providing hands-on instruction may therefore be as important as increasing ownership rates.

The present study found that education level was a consistent predictor of awareness, trust, and child car seat use, while socioeconomic status (SES) was not a strong direct predictor of use. However, SES strongly influenced support for subsidization policies, with lower-income parents significantly more likely to support financial assistance for child car seats. At the same time, the strong association between income level and support for subsidies highlights the role of economic constraints in shaping safety behaviors and policy preferences. While knowledge and motivation may exist across income groups, affordability remains a structural barrier for many families. This raises important equity considerations, as child safety interventions that rely solely on voluntary purchase may disproportionately benefit higher-income families. The literature increasingly emphasizes that equitable road safety policies must address both knowledge gaps and financial barriers to ensure that all children benefit from protective measures [37,38].

Parents in this study expressed overwhelming support for subsidized child car seats (92%), along with strong endorsement of media campaigns (88%), workshops at health centers (84%), and stricter enforcement (78%). Importantly, these preferences closely mirror the barriers identified in both quantitative and qualitative analyses, indicating a strong alignment between perceived problems and desired solutions. Similar findings have been reported in prior research, where parents favored interventions that reduced financial burden and improved practical knowledge rather than punitive approaches alone [39].

The high level of support for education delivered through health centers underscores the role of trusted institutions in promoting child safety behaviors. Health professionals are consistently identified as credible sources of information, and integrating child passenger safety education into routine health services has been shown to improve both use and correct installation of child restraints. Furthermore, the substantial support for enforcement suggests public readiness for stronger policy measures, provided they are accompanied by education and access to affordable equipment. These findings reinforce the interpretation that parents are not resistant to safety, but rather constrained by systemic, cultural, and economic factors.

The use of convenience sampling through public health centers may restrict the generalizability of the findings to all parents in Tabriz or other regions of Iran; however, inclusion of multiple centers from different geographic areas of the city helped increase sample diversity. In addition, the reliance on self-reported measures may have introduced recall bias or social desirability bias, potentially resulting in overreporting of safe practices. Future studies are recommended to adopt longitudinal or interventional designs to better clarify causal relationships and assess the impact of targeted educational or policy-driven strategies, and to utilize probability-based sampling approaches or conduct multi-city investigations to strengthen representativeness and external validity.

## Conclusion

This study explores the aspects of awareness, trust, culture, and behavioral practices of parents regarding the use of car seats for children. Among the participants, even though there was a high rate of accessibility to private vehicles, child car seats were not owned and used frequently. This reveals an important gap regarding safety behavior and the resources available. It was demonstrated that there were a great number of parents who knew about the protective benefits that a child car seat brings, though this knowledge rarely resulted in positive outcomes when it came to actual proper and continuous use, especially for short travel within the city. Primary use and correct use of safety seats were greatly influenced by the participant’s trust in the safety recommendations and the qualitative worth of the car safety seats. Values and beliefs on risk including social and cultural influences, underestimating risk, and traditional practices further speculated incidents that put children at risk like carrying children and not putting on seat belts. A major concern that was conveyed by the focus of using a child car safety was not using the car seat properly. This posed a potential risk of lesser marginal benefit of the child safety seat when used poorly. Parents expressed interests in public awareness campaigns and subsidized child car safety seats available to them. This study demonstrates that parents want their children to be safe in a vehicle, but they are highly influenced by the cost, social pressures, and actual safety of the child restraint system. In conclusion, improving child passenger safety requires moving beyond awareness-raising alone. Policymakers and public health practitioners should implement integrated, multilevel strategies that address affordability, practical skills, product trustworthiness, cultural norms, and enforcement. Such approaches are essential to ensure not only the adoption but also the correct and consistent use of child car seats, ultimately reducing preventable injuries among young children.

## Supporting information

S1 File Dataset for analysis. (XLSX)
